# Supplementary material for: Piezomagnetoelectric effects in a candidate Kitaev magnet
Source: Nat Commun. 2026 May 30;17:4860. doi: 10.1038/s41467-026-73747-0 (PMC13226674; doi:10.1038/s41467-026-73747-0)
Supplement: Supplementary file 1 — Supplementary Information [file 41467_2026_73747_MOESM1_ESM.pdf]

Supplementary Information:
Piezomagnetoelectric effects in a candidate Kitaev magnet

Vilmos Kocsis,[1] Sven Luther,[2] Nicolás Pérez,[1] Weiliang Yao,[3]
Hannes Kühne,[2] Anja U. B. Wolter,[1] Yuan Li,[4,3] and Bernd Büchner[1,5,6]

[1] *Institut für Festkörperforschung, Leibniz IFW Dresden, 01069 Dresden, Germany*
[2] *Hochfeld-Magnetlabor Dresden (HLD-EMFL), Helmholtz-Zentrum Dresden-Rossendorf, 01328 Dresden, Germany*
[3] *International Center for Quantum Materials, School of Physics, Peking University, 100871 Beijing, China*
[4] *Beijing National Laboratory for Condensed Matter Physics, Institute of Physics, Chinese Academy of Sciences, 100190 Beijing, China*
[5] *Institute of Solid State and Materials Physics and Würzburg-Dresden Cluster of Excellence ctd.qmat, Technische Universität Dresden, 01062 Dresden, Germany*
[6] *Center for Transport and Devices, Technische Universität Dresden, 01069 Dresden, Germany*

The supplementary information contains additional magnetization, heat capacity, thermal expansion, and magnetostriction measurements.

Figure S1 shows the high-field magnetization measurements up to $\mu_0 H$=50 T fields. The pulsed-field measurement were done at T=1.4 K in the presence of $\mathbf{H} \parallel a$, $\mathbf{H} \parallel a^*$, and $\mathbf{H} \parallel c$ fields. In Fig. S1(a), we show the directly measured dM/dH curves, while in Fig. S1(b) we compare the integrated magnetization curves to those of the dc measurements. The dc magnetization curves were measured at T=2.0 K up to $\mu_0 H$=14 T fields using the QD-PPMS VSM option. The high-field magnetization measurements reveal no further phase transitions above H=95 kOe. Here, we assume that the source of the weak ferromagnetic moment is well described by spin-canting. Using the combination of the low- and high-field measurements, we can calculate an estimation for the canting angle: The reduced magnetization for H=5 kOe is $M - \chi \cdot H \approx 0.13 \pm 0.2\,\mu_B$/f.u., while the saturated moment is estimated to $M^{\mathrm{Sat}} \approx 5.5\,\mu_B$/f.u., which gives a ϑ=1.3 deg canting angle.

Figures S2 and S3 show additional C_p heat capacity, α_c thermal expansion, and λ_c magnetostriction measurement data related to the phase diagram in Fig. 4(a), $\mathbf{H} \parallel a^*$.

In Fig. S4, we compare additional λ_c magnetostriction measurements performed on three different samples at T=8 K for $\Delta L_c \parallel c$ and $\mathbf{H} \parallel a^*$. In Fig. S4(a), we repeat the λ_c-H data shown in Fig. 2(d) measured on the 190 μm thick sample. In Fig. S4(b) and S4(c), we show the λ_c-H data measured on a 280 μm and a 30 μm thick sample pieces, respectively. The reproducibility of the magnetostriction measurements is remarkably high, and detailed features in the λ_c-H data are well reproduced across all three samples, including the overall magnitudes of the peaks in λ_c. Most remarkably, although the signal-to-noise ratio is worst for the extremely thin 30 μm sample, we find that all phase transitions are reproduced within the measurement accuracy. However, when the two thickest samples are stacked on top of each other, as shown in Fig. S4(d), only the most prominent features at H=0 kOe and H=±55 kOe are reproduced and finer details are lost. This difference may be related to slight sliding of the samples on each other, or a compensation effect of shearing deformations. We note that a similar stacking of crystals for dilatometry measurements was applied in Ref. [1].

Figure S5 discusses the poling field dependence of the magnetoelectric properties of $Na_2Co_2TeO_6$. Similarly to the P-H curves in the main text, prior to every single measurement the sample was warmed to T=50 K, then cooled to the ordered phase in the presence (or absence) of $\mathbf{E}_0 \parallel a$ and $\mathbf{H}_0 \parallel a^*$ poling fields. Following the poling, the electric field was removed and the leads were shorted for 10-minute-long intervals for a dozen times for electrostatic discharge, then the P was measured at T=8.0 K in sweeping H field starting from H_0. As discussed in the main text, when finite E_0=±1.5 kV/cm and H_0=±50 kOe fields are applied the P-H curves show high susceptibility and a large jump at H=55kOe. We also find a similarly large susceptibility when no electric field but the same magnetic field is applied during the poling procedure, namely E_0=0 kV/cm and H_0=±50 kOe. The ME properties noticeably change when the poling magnetic field is changed to H_0=+90 kOe or H_0=0 kOe. When the sample is zero-field-cooled (E_0=0 kV/cm, H_0=0 kOe), the P-H curve is symmetric in H field and the jump at H=55kOe is much reduced.

In Fig. S7, we discuss the hysteresis appearing in the magnetization and magnetostriction measurements. In Fig. S7(a), we show the magnetization measured at T=8 K for the field up (↑, yellow curve) and down runs (↓, blue curve), while the hysteresis is gauged by the difference, defined as $\delta M = (M_\downarrow - M_\uparrow)/2$ (green curve, scaled for better visibility). The hysteresis is largest at H=0 kOe and H=±55 kOe, due to the weak ferromagnetism and the spin-flop transition, respectively, and completely disappears above H=±60 kOe. In Fig. S7(b), the magnetostriction measurements show a different picture. The hysteresis in the magnetostriction, which is defined similarly to that of the magnetization, $\delta L/L_0 = (\Delta L/L_{0,\downarrow} - \Delta L/L_{0,\uparrow})/2$, shows zero hysteresis at H=0 kOe. This means that the

deformation is elastic and the crystal fully returns to its original elastic state when the field is removed, and the hysteretic part of the deformation is linear in magnetic field. A possible interpretation is illustrated in Fig. S7(c). In the hexagonal and piezo-magnetoelectric P$6_3$2'2' magnetic space group, shearing deformations are allowed with the linear piezomagnetic-effect: $\epsilon_{a^*c} = \lambda_{a^*ca^*} H_{a^*}$, where ϵ_{a^*c} is the a^*c component of the $\hat{\epsilon}$ deformation tensor, $\lambda_{a^*ca^*}$ is one of the allowed piezomagnetic tensor elements, and H_{a^*} is the a^* component of the magnetic field. The ϵ_{a^*c} means such a deformation, where the top and bottom aa^* planes of the unit cell are slid along the a^* axis, which deformation component tensor element is not expected to be detected by our dilatometry measurements with $\Delta L_c \parallel c$. This is because in general $\Delta L = \mathbf{n}\hat{\epsilon}\mathbf{n}$, where $\mathbf{n}$ is the axis of the dilatometry measurement. However, the case of $Na_2Co_2TeO_6$ is particular, due to the distribution of the Na^+ ions within the unit cell. As a possible scenario, the shearing ϵ_{a^*c} deformation induced by the H_{a^*} magnetic field slides the Na^+ ions within the hexagonal plane, and redistribute them more evenly or slide them on top of each other, as illustrated in Fig. S7(c) with figures (1) to (4). Therefore, the redistribution of the Na^+ ions along the c axis caused by the shearing component of the linear piezomagnetic effect may provide an explanation for the observed hysteresis in our magnetostriction measurements. We note, that the displacement of the Na^+ ions may also explain the hysteresis in the P-H and dielectric constant curves, however, further measurements would be needed to confirm this explanation.

The requirements for the ME effect and the pristine Kitaev interaction are considered mutually excluding. This greatly limits the number of QSL candidates where we can expect ME effects, as we list several QSL candidate materials in Tab. S1. However, each of these contradictions has a resolution. As an example, the ME effect is usually considered in materials with long-range order, while the QSL ground state of the pristine Kitaev model avoids traditional long-range order. However, ME materials with the *pd*-hybridization mechanism are exceptional in this sense, as it can lead to ME effect even in the absence of long-range order [2]. As a second example, in regards of the Kitaev model ME effect is not expected, as in a S=1/2 system described by spin polymatrix models the higher order $S_{i,\mu}S_{i,\nu}$, μ,ν=x,y,z on-site terms in the Hamiltonian are forbidden. These higher order on-site terms are in general required for the theoretical description of ME effect originated from the *pd*-hybridization mechanism. However, in real-life S=1/2 materials, such as in $Na_2Co_2TeO_6$ and $CoSe_2O_5$ [3], these terms can be still allowed, particularly if the local environment of the magnetic ion is distorted. The only requirement for the coexistence of the ME effect with the Kitaev interaction is that the distortion of the ligand octahedron has to be still compatible with the Kitaev interaction, such as trigonal distortion. Moreover, the off-diagonal Γ, Γ' terms in the Heisenberg-Kitaev model may also lead to the ME effect due to the spin-current mechanism. Still, in the list of materials in Tab. S1, apart from the ME triple-q phase of $Na_2Co_2TeO_6$ we find only the trigonal structural phase of α-$RuCl_3$ to possibly have a polar magnetic ground state, similarly to $PbCuTe_2O_6$.

Here we note, that while Ref. 4 reports the emergence of a ferroelectric order at T_{FE} ~70 K, similarly to Refs. 5 and 6 we do not find evidence for any ferroelectricity at this temperature range (see Fig. S6).

[1] M. Frachet, L. Wang, W. Xia, Y. Guo, M. He, N. Maraytta, R. Heid, A.-A. Haghighirad, M. Merz, C. Meingast, and F. Hardy, PRL **132**, 186001 (2024).

[2] M. Akaki, H. Kuwahara, A. Matsuo, K. Kindo, and M. Tokunaga, Journal of the Physical Society of Japan **83**, 093704 (2014).

[3] L. Lin, Y. S. Tang, L. Huang, W. J. Zhai, G. Z. Zhou, J. H. Zhang, M. F. Liu, G. Y. Li, X. Y. Li, Z. B. Yan, and J.-M. Liu, Appl. Phys. Lett. **120**, 052901.

[4] S. Mukherjee, G. Manna, P. Saha, S. Majumdar, and S. Giri, Phys. Rev. Mater. **6**, 054407 (2022).

[5] S. Zhang, S. Lee, A. J. Woods, W. K. Peria, S. M. Thomas, R. Movshovich, E. Brosha, Q. Huang, H. Zhou, V. S. Zapf, and M. Lee, Phys. Rev. B **108**, 064421 (2023).

[6] C. Dhanasekhar, M. Jawale, R. Kumar, D. C. Kakarla, S. Mahapatra, S. Kaushik, P. Babu, M. M. Chou, A. Sundaresan, H. Yang, and A. Mahajan, Chinese Journal of Physics **98**, 36 (2025).

[7] C. Ritter, Journal of Physics: Conference Series **746**, 012060 (2016).

[8] H. B. Cao, A. Banerjee, J.-Q. Yan, C. A. Bridges, M. D. Lumsden, D. G. Mandrus, D. A. Tennant, B. C. Chakoumakos, and S. E. Nagler, PRB **93**, 134423 (2016).

[9] Y. Imai, K. Nawa, Y. Shimizu, W. Yamada, H. Fujihara, T. Aoyama, R. Takahashi, D. Okuyama, T. Ohashi, M. Hagihala, S. Torii, D. Morikawa, M. Terauchi, T. Kawamata, M. Kato, H. Gotou, M. Itoh, T. J. Sato, and K. Ohgushi, Phys. Rev. B **105**, L041112 (2022).

[10] A. Biffin, R. D. Johnson, S. Choi, F. Freund, S. Manni, A. Bombardi, P. Manuel, P. Gegenwart, and R. Coldea, Phys. Rev. B **90**, 205116 (2014).

[11] S. C. Williams, R. D. Johnson, F. Freund, S. Choi, A. Jesche, I. Kimchi, S. Manni, A. Bombardi, P. Manuel, P. Gegenwart, and R. Coldea, Phys. Rev. B **93**, 195158 (2016).

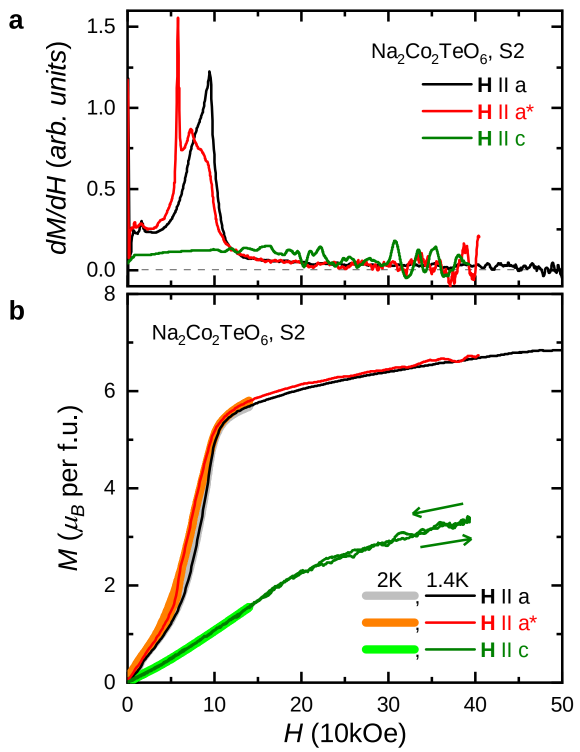

FIG. S1. **Pulsed-field magnetization measurements.** High-field magnetization measurements up to $\mu_0 H$=50 T were done in pulsed magnetic fields. (a) The dM/dH curves were measured directly at T=1.4 K for $\mathbf{H} \parallel a$, $\mathbf{H} \parallel a^*$, and $\mathbf{H} \parallel c$. (b) The integrated dM/dH pulsed field measurements compared to the dc magnetization curves.

[12] F. Ye, S. Chi, H. Cao, B. C. Chakoumakos, J. A. Fernandez-Baca, R. Custelcean, T. F. Qi, O. B. Korneta, and G. Cao, Phys. Rev. B **85**, 180403 (2012).

[13] E. Lefrançois, M. Songvilay, J. Robert, G. Nataf, E. Jordan, L. Chaix, C. V. Colin, P. Lejay, A. Hadj-Azzem, R. Ballou, and V. Simonet, PRB **94**, 214416 (2016).

[14] W. Chen, X. Li, Z. Hu, Z. Hu, L. Yue, R. Sutarto, F. He, K. Iida, K. Kamazawa, W. Yu, X. Lin, and Y. Li, PRB **103**, L180404 (2021).

[15] J.-Q. Yan, S. Okamoto, Y. Wu, Q. Zheng, H. D. Zhou, H. B. Cao, and M. A. McGuire, Phys. Rev. Mater. **3**, 074405 (2019).

[16] P. K. Mukharjee, B. Shen, S. Erdmann, A. Jesche, J. Kaiser, P. R. Baral, O. Zaharko, P. Gegenwart, and A. A. Tsirlin, Intermediate field-induced phase of the honeycomb magnet baco$_2$(aso$_4$)$_2$ (2024), arXiv:2403.04466 [cond-mat.str-el].

[17] C. Thurn, P. Eibisch, A. Ata, M. Winkler, P. Lunkenheimer, I. Kézsmárki, U. Tutsch, Y. Saito, S. Hartmann, J. Zimmermann, A. R. N. Hanna, A. T. M. N. Islam, S. Chillal, B. Lake, B. Wolf, and M. Lang, npj Quantum Materials **6**, 95 (2021).

[18] S. V. Gallego, J. M. Perez-Mato, L. Elcoro, E. S. Tasci, R. M. Hanson, K. Momma, M. I. Aroyo, and G. Madariaga, Journal of Applied Crystallography **49**, 1750 (2016).

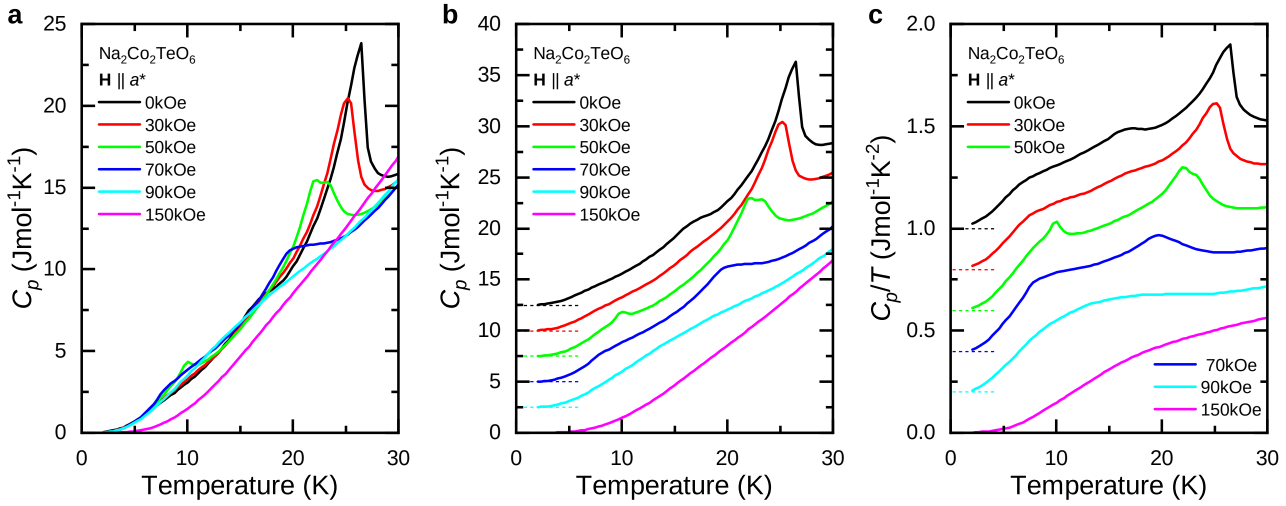

FIG. S2. **Heat capacity measurements.** (a) Temperature dependence of the C_p heat capacity measured in the presence of $\mathbf{H} \parallel a^*$ field, shown for selected fields. (b) Heat capacity data shown with uniform stacking along the vertical axis for better visibility. (c) Temperature dependence of the C_p/T with uniform stacking along the vertical axis for better visibility.

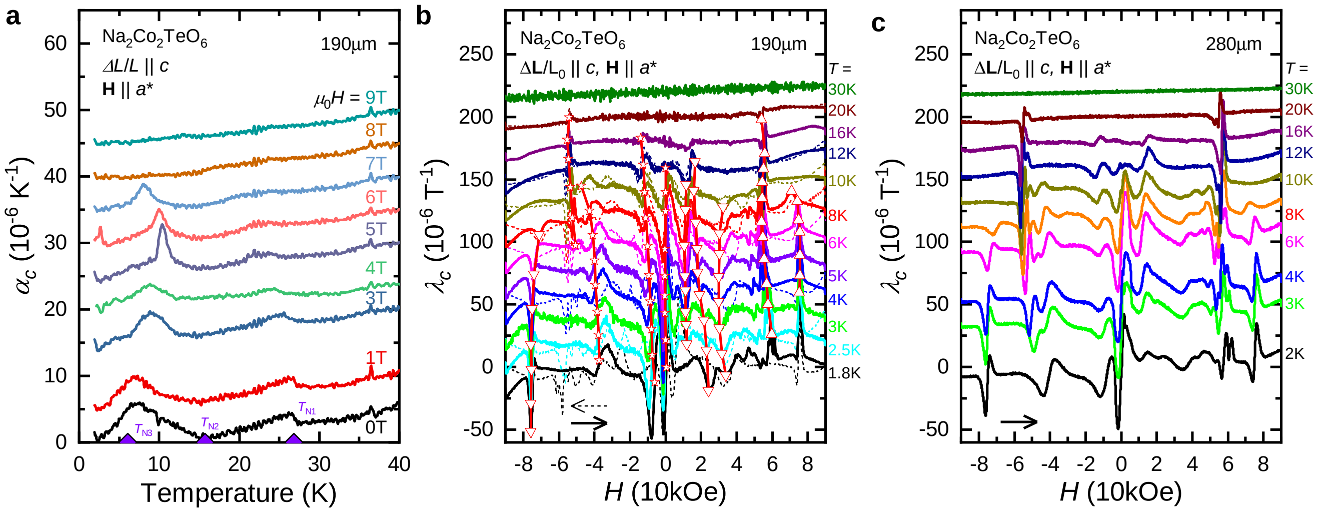

FIG. S3. **Temperature and magnetic field dependent α_c thermal expansion and λ_c magnetostriction measurements.** (a) Temperature dependence of the α_c thermal expansion coefficient measured for $\Delta L_c \parallel c$ and shown for selected $\mathbf{H} \parallel a^*$ magnetic fields. For better visibility the α-T curves are stacked along the vertical axis. (b-c) Magnetic field dependence of the λ_c magnetostriction measured for $\Delta L_c \parallel c$ at selected temperatures in $\mathbf{H} \parallel a^*$ magnetic fields measured for the 190 μm and the 280 μm thick samples, shown in panels (b) and (c), respectively. Solid and dashed curves correspond to measurements in increasing and decreasing fields, respectively. Symbols denote the features associated with the phase transitions shown in Fig. 4(a).

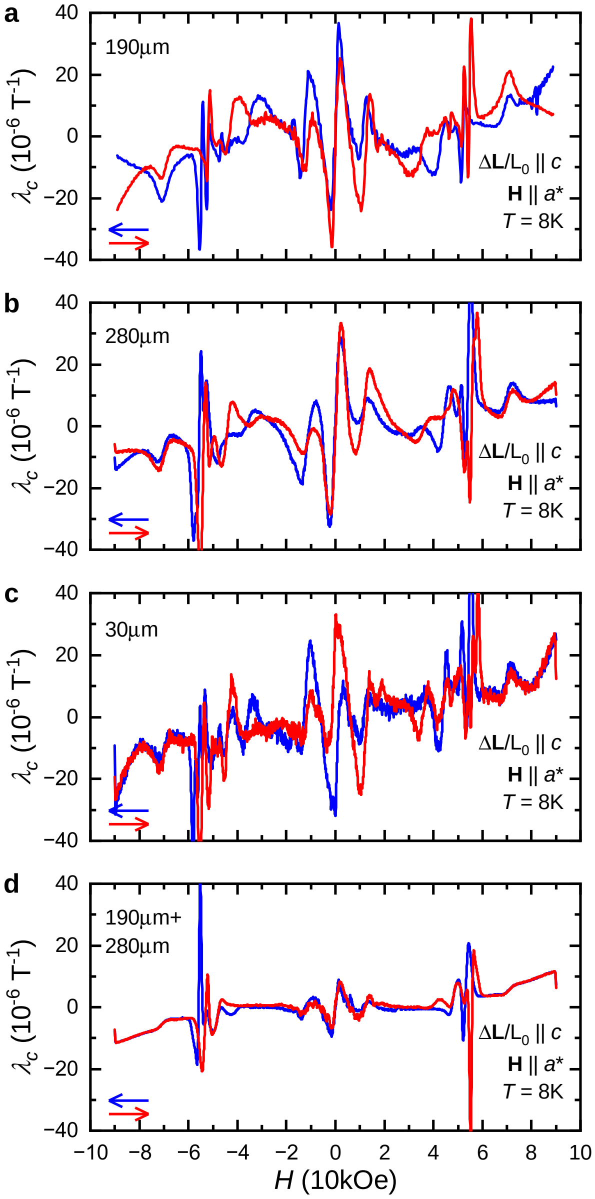

FIG. S4. **Sample dependence of the λ_c-H magnetostriction measurements.** In panels (a) to (c) we show λ_c-H magnetostriction measurements performed on a 190 μm (same data as in the main text), a 280 μm, and an extremely thin 30 μm sample, respectively. In panel (d), we show λ_c-H magnetostriction measurements when the two thickest samples are stacked on top of each other.

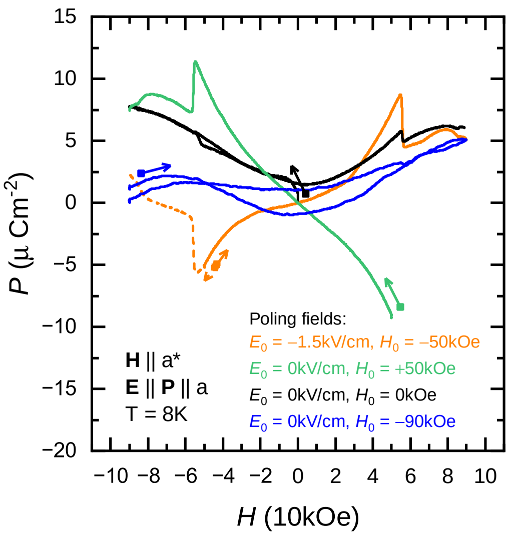

FIG. S5. **Poling-field dependence of the P-H curves.** The H-field induced P polarization measured at T=8 K is shown for selected poling fields. The sample was cooled below T_{N1} in the presence or absence of $\mathbf{H} \parallel a^*$, $\mathbf{E} \parallel a$ poling fields. In the absence of appropriate poling, the P-H curves show only a minor change at the spin-flop like phase transition at H=55 kOe.

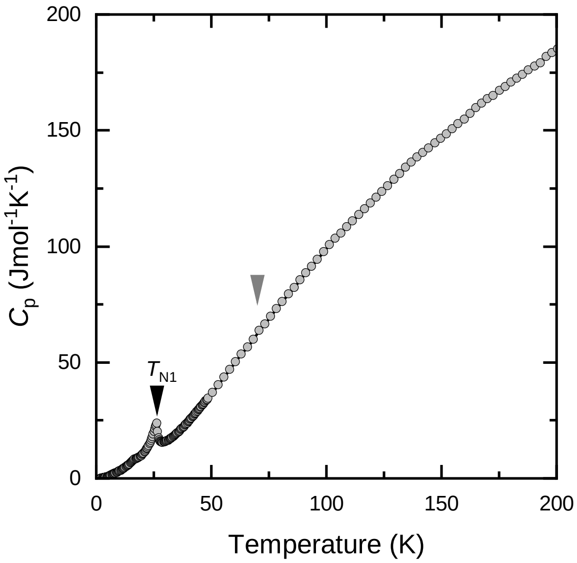

FIG. S6. **Heat capacity measurements up to T = 200 K.** The sample shows anomalies at the magnetic phase transitions, T_{N1}, T_{N2}, and T_{N3}, but we find no anomaly around 70 K (indicated by grey triangle) where a suggested ferroelectric phase transition was reported.

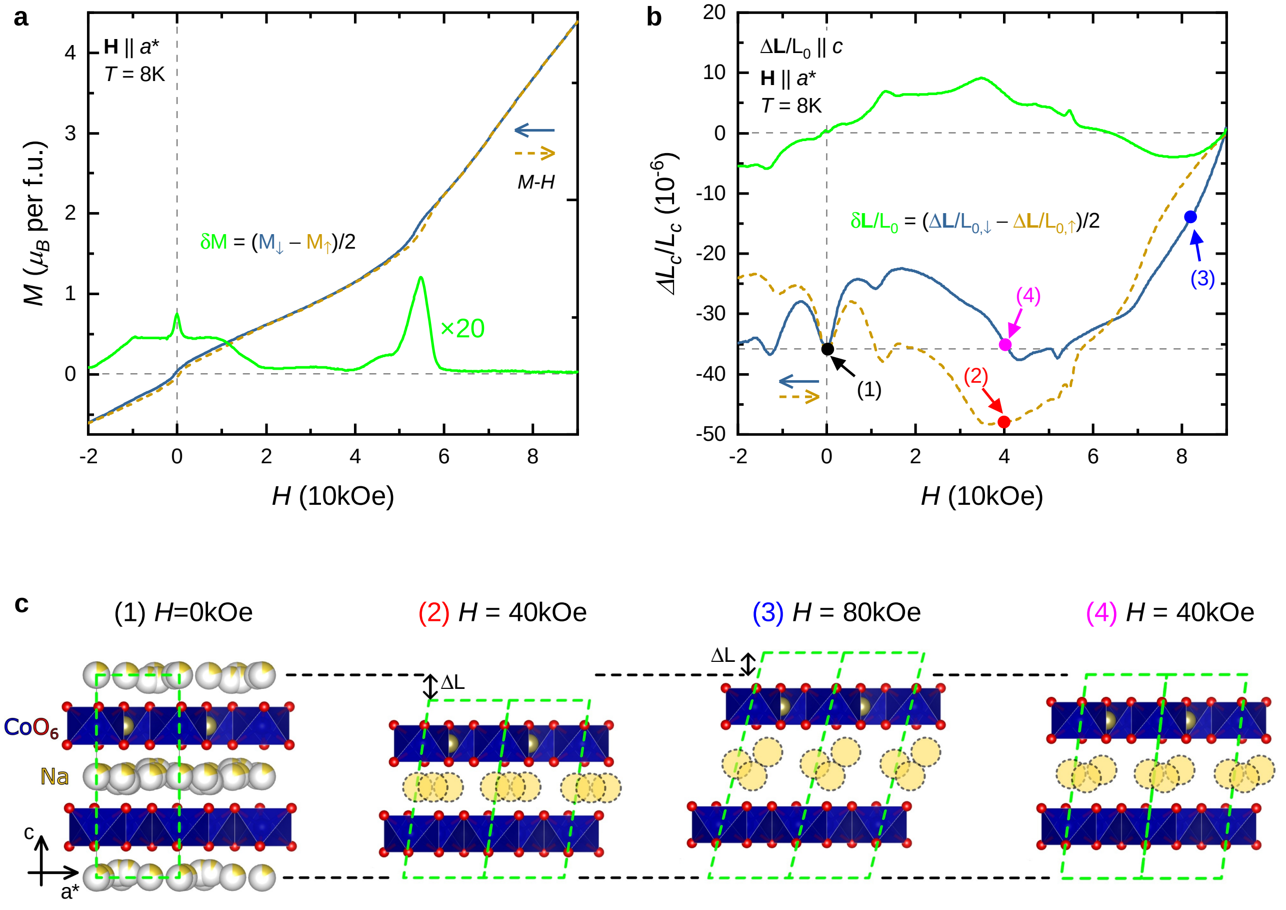

FIG. S7. **Hysteresis in the magnetization and magnetostriction measurements.** Magnetic field dependence of the magnetization (a) and the $\Delta L/L_0$ relative length change at T=8 K, $\Delta L \parallel c$ and $\mathbf{H} \parallel a^*$. The hysteresis in both M and $\Delta L \parallel c$ are represented by the difference between the field up (↑) and field down (↓) runs, $\delta M = (M_\downarrow - M_\uparrow)/2$ and $\delta L/L_0 = (\Delta L/L_{0,\downarrow} - \Delta L/L_{0,\uparrow})/2$, respectively. The hysteresis curve form the magnetization (δM, green curve) in panel (a) is scaled by a factor of 20 for better visibility, while the hysteresis curve of the relative length change in panel (b) $\delta L/L_0$ is unscaled. In panel (c), we illustrate a possible explanation for the observed large hyseresis in the relative length change, due to the redistribution of the Na^+ ions along the c axis caused by the $\epsilon_{\epsilon_{a^*c}}$ shearing-deformation of the linear piezomagnetic effect. Sub-figures (1) to (4) illustrate specific deformed states corresponding to the labels in panel (b).

Material	Magn. order	Struct.	m.s.g. (m.p.g.)	MX_6	Polar	ME	piezoME
α-$RuCl_3$ [7]	q=(1/2, 0, 1/2)	$P3_112$	$C_{2C}2$ (2.1')	slight dist.	**yes**	no	no
α-$RuCl_3$ [8]	q=(0, 1, 1/3)	C2/m	C_P2/m (2/m.1')	slight dist.	no	no	no
$RuBr_3$ [9]	q=(0, 1/2, 1)	R-3	P_{2S}-1 (-1.1')	slight dist.	no	no	no
Li_2IrO_3 [10]	q=(0.577, 0, 0)	Fddd	Fddd1' (mmm.1')	slight dist.	no	no	no
Li_2IrO_3 [11]	q=(0.319, 0, 0)	C2/m	C2/m1' (2/m.1')	slight dist.	no	no	no
Na_2IrO_3 [12]	q=(0, 1, 1/2)	C2/m	$C_{2C}2/m$ (2/m.1')	slight dist.	no	no	no
$Na_2Co_2TeO_6$ [13]	q=(1/2, 0, 0)	$P6_322$	$C_P2'2'2_1$ (222.1')	strong dist.	no	no	no
$Na_2Co_2TeO_6$ [14]	tripple-q	$P6_322$	$P6_32'2'$ (62'2')	strong dist.	no	**yes**	**yes**
$Na_3Co_2SbO_6$ [15]	q=(1/2, 1/2, 0)	C2/m	P_{2S}-1 (-1.1')	strong dist.	no	no	no
$BaCo_2(AsO_4)_2$ [16]	uudd	R-3	-	regular	no	-	-
$PbCuTe_2O_6$ [17]	no (20 mK<)	$P4_132$	-	n.a.	**yes**	no	no
$CoSe_2O_5$ [3]	q=(0, 0, 0)	Pbcn	Pb'cn (m'mm)	strong dist.	no	**yes**	**yes**

TABLE S1. **Kitaev QSL candidate materials compatibility for hosting ferroelectricity, ME effect, and piezomagnetoelectricity.** Known magnetic orders were taken from literature and MAGNDATA [18] of the Bilbao Crystallographic Server. The table only considers the magnetic ground states. The *uudd* labels an up-up-down-down order. Magnetic space group (m.s.g.) names are shown in the Opechowski & Guccione setting, while we indicate magnetic point group (m.p.g.) in brackets. While not Kitaev QSL candidates, we added the polar QSL $PbCuTe_2O_6$ and the spin chain $CoSe_2O_5$ as references.
